# Emulsified silicone oil is taken up by and induces pro-inflammatory response in primary retinal microglia

**DOI:** 10.1007/s00417-020-04763-z

**Published:** 2020-06-04

**Authors:** Alexa Klettner, Antonia Harms, Vicki Waetzig, Jan Tode, Konstantine Purtskhvanidze, Johann Roider

**Affiliations:** 1 Department of Ophthalmology, University Medical Center, University of Kiel, Arnold-Heller-Str. 3, Haus 25, 24105 Kiel, Germany; 2 Department of Pharmacology, University Medical Center, University of Kiel, Hospitalstr. 4, 24105 Kiel, Germany

**Keywords:** Silicone oil, Endotamponade, Microglia, IL-6, IL-8

## Abstract

**Purpose:**

Silicone oil is used as endotamponade in combination with vitrectomy. Thinning of retinal layers and loss of retinal cells under silicone oil use have been found. Here, we investigate the influence of silicone oil on primary microglia cells.

**Methods:**

Primary microglia cells were prepared from the porcine retina. Microglia identity was assessed with Iba1 staining. Silicone oil was emulsified by sonification. Cell morphology and silicone oil uptake were evaluated by light microscopy after Coomassie blue staining. Cytokine secretion was evaluated with ELISA. Toxicity of silicone oil on microglia and toxic effect of silicone oil–treated microglia on neuronal cell line PC12 were evaluated by MTT or WST assay, respectively.

**Results:**

Microglia took up silicone oil droplets after 72 h of incubation. Silicone oil induced no toxicity but increased the metabolism in microglial cells. In addition, the secretion of IL-6 and IL-8, but not of IL-1ß or TNF-α, was induced. Silicone oil–treated microglia did not exert any neurotoxic effect on differentiated PC12 cells but induced an increase in metabolism.

**Conclusion:**

Emulsified silicone oil changes the activity level of microglia and induces the secretion of IL-6 and IL-8. Neurotoxicity is not induced. Further experiments are required to investigate the long-term effect of silicone oil on microglia and their consequent effect on neuronal cells.

## Introduction

In the management of severe vitreoretinal diseases such as retinal detachment with proliferative vitroretinopathy, giant retinal tear, or multiple retinal tears, silicone oil is widely used as long-term endotamponade.

Due to stability, immunological tolerability, and lack of direct toxicity, it is generally regarded as a safe compound and can therefore be used as a long-acting tamponade [1, 2]. Silicone oil has been in clinical use for more than 30 years, mostly in complicated cases or in reoperations. A variety of complications have been described for the use of silicone oil, including cysts, emulsification, and migration of the oil [3]. Therefore, it is recommended to remove silicone oil within a period of 3 months up to 1 year. Recently, severe loss of visual acuity has been—initially anecdotally—reported in individual cases. Detailed studies and studies on a very well-documented patient population indicate that a severe loss in visual acuity might occur much more frequently than generally assumed. They showed that in 20–30% of patients treated with silicone oil visual acuity is much worse than could be expected [4, 5]. In such cases, all the clinical investigations, e.g., fluorescein angiography, OCT, or analysis of the operating report, did not give a satisfactory explanation for the decline in visual acuity. Tode et al. have shown that this unexplained loss was accompanied by a thinning of foveal and parafoveal combined nerve fiber, ganglion cell, and inner plexiform layers.

The mechanisms behind this pathology are not known so far. In earlier studies, silicone oil–associated vision loss was discussed to be associated with silicone oil droplets found in the retina and optic nerve [6]. Silicone oil tends to emulsify in retinal tissue as saccadic, and pursuit eye and head movements cause shear stress on silicone oil bubbles [7]. While these bubbles are generally considered to be transient, they may become permanent in the presence of blood components or pro-inflammatory proteins [2]. Contrary to the immunological tolerability attributed to silicone oil, emulsified silicone oil bubbles are suspected to trigger inflammation, phagocytosis, and chemotaxis [2]. These cases usually have a history of complicated surgery or pathology. However, the causes of vision loss in cases with or without such a history are not known so far. Roider et al. postulated some immunological reactions in the fovea due to the exposure of microglial cells to silicone oil (VAIL-Vitrectomy 2016).

Silicone oil has previously been described to be incorporated by macrophages, which was linked to transport of silicone oil into the retina, optic nerve, or brain [8–10]. Blood-borne inflammatory-activated macrophages are found in epiretinal membranes of silicone oil–treated eyes but hardly in the corresponding retina [11]. This correlates well to the fact that the innate immune cell of the retina is the microglia, not the macrophage. Microglia are not blood-borne but migrate into the retina from the yolk sac during embryogenesis [12]. In healthy retinas, microglia are located in the inner retinal layers, constantly surveying their surroundings, while upon activation, they become motile, pro-inflammatory, and phagocytic. Involvement of microglia in retinal degenerative diseases is widely discussed especially as prolonged activated microglia may become neurotoxic [13]. Microglial interaction with silicone oil has not been investigated so far. In this study, we have treated primary retinal microglia with emulsified silicone oil, investigating toxicity, morphology, and pro-inflammatory cytokine release.

## Materials and methods

### Microglia cells

Primary microglia were prepared from the porcine retina as described [14]. For experimentation, cells were harvested and seeded at a cell density of 250,000 cells/well in 24-well (Sarstedt, Nümbrecht, Germany) or 96-well plates (Roth, Karlsruhe, Germany). Cell number was assessed with trypan blue exclusion assay. Twenty-four hours after seeding, the medium was changed and the appropriate substance was added. Emulsified silicone oil (1%, 5%, 10%; Oxane 5700, Bausch & Lomb GmbH, Berlin, Germany) was incubated for 24–72 h. Polyinosinic:polycytidylic acid (Poly I:C; Tocris, Bristol, UK; 100 μg/ml) was used as a positive control for microglia stimulation [15].

### PC12 cells

Differentiated rat pheochromocytoma PC12 cells are a widely used cell culture model for neuronal cells [16], especially concerning neurotoxicity and neuroprotection [17]. These cells were directly obtained from the German Collection of Microorganisms and Cell Cultures (DSMZ), Braunschweig, Germany. No subclones were used. Cells were cultured on collagen-coated plates in RPMI 1640 medium (Life Technologies; Darmstadt, Germany) supplemented with 5% fetal bovine serum (FBS; Biochrom; Berlin, Germany) and 10% horse serum (Biochrom) at 37 °C and 5% CO_2_. For neuronal differentiation, PC12 cells were plated at low density and kept in serum-containing medium for 24 h, cultured in medium supplemented with 0.5% FBS and 1% penicillin/streptomycin for additional 72 h, and treated with nerve growth factor (NGF) (50 ng/ml; Alomone Labs; Jerusalem, Israel) for seven days. Fresh NGF was added every second day [18].

### Fluorescence microscopy

Iba1 is considered a marker for microglial cells [13]; therefore, Iba1 staining was conducted to assess the purity of the cells as previously described [14]. Per slide, eight pictures were taken and cell nuclei and Iba1 fluorescent cells were assessed. The number of nuclei was set in relation to Iba1 positive-stained cells.

### Emulsification of silicone oil

Silicone oil was added in a concentration of 1%, 5%, or 10%, respectively, in cell culture medium (DMEM, supplemented with 10% FBS and 1% penicillin/streptomycin) and vortexed. The mixture was sonicated for 30 min in a sonication bath (Ultrasonic Cleaner USC-TH VWR, Darmstadt). Bubble formation was verified in light microscopy.

### Coomassie staining

For light microscopic evaluation of morphology and silicone oil uptake, cells were stained with Coomassie. After incubation and removal of the supernatant, cells were washed with PBS and fixed for 40 min with 2.5% glutaraldehyde (Merck, Darmstadt, Germany). Cells were washed with PBS and stained for 10 min with Coomassie Brilliant Blue R-250 staining solution (Bio-Rad, München, Germany) and destained for 2 × 20 min with Coomassie Brilliant Blue R-250 destaining solution (Bio-Rad). Afterwards, cells were washed three times with PBS and once with Aqua Dest (Ampuwa, Fresenius, Bad Homburg, Germany) and mounted with Faramount (Dako, Hamburg, Germany).

### Microglia morphology

Microglia were categorized according to morphological features [19]. “Resting” microglia presented as small cells (100–500 μm^2^) with many ramifications. “Ameboid” cells presented as larger (500–1000 μm^2^), plump cells that had lost their ramification. A third category is the large “giant” cells [20], with a size above 1000 μm^2^ and polymorphic cell shape.

### Silicone oil inclusions

Coomassie-stained microglia were analyzed in light microscopy. For all cover slips, nine pictures were taken in a fixed pattern and the silicone oil droplets per cell were assessed. A silicone oil droplet had to present as a clearly bordered intracellular inclusion that was devoid of blue staining (× 20 magnification). Inclusions were not regarded as silicone oil droplets if they did not present with a clear border to the adjunct cell plasma.

### MTT assay

In order to test the direct toxicity of silicone oil on the microglia cells, an MTT assay (3-(4,5-dimethylthiazol-2-yl)2,5diphenyltetrazoliumbromid) was conducted [21]. Cells were incubated with different concentrations of emulsified silicone oil for 24, 48, or 72 h and incubated with MTT (Sigma-Aldrich, Steinheim, Germany) in DMEM (without phenol red) for 2 h. Supernatant was discarded, and DMSO (Roth) was added to the cells and rocked. The extinction of the supernatant was measured at a wavelength of 550 nm (ELx800, BioTek, Bad Friedrichshall, Germany), and DMSO served as a blank control. Untreated cells served as control and were set at 100%.

### WST assay

To test the toxic effects of the supernatant of microglial cells on PC12 cells, a WST (water-soluble tetrazolium) assay was conducted. In this test, the activity of the superoxide dismutase is detected, as a surrogate parameter for cell activity [22]. Cells were grown on 96-well plates (2 × 10^6^ cells per well) and treated for 48 h with the supernatant of microglia that had been treated with emulsified silicone oil for 72 h. As respective controls, PC12 cells were treated with supernatant of untreated microglia, emulsified silicone oil without microglia medium, or medium without further treatment. After the experiment, the medium was discarded and 10 μl of water-soluble tetrazolium-1 (WST-1) reagent (Sigma; Munich, Germany) was added to each well to determine metabolic activity. The cells were incubated for 2 h at 37 °C and 5% CO^2^. The plate was placed into a Tecan infinite M200 reader (Tecan; Crailsheim, Germany) and shaken thoroughly for 1 min. Absorbance was measured at 450 nm with a reference wavelength of 600 nm. Per plate, one well with cells treated only with medium without further treatment was set as 100%, and the other values were calculated accordingly.

### Enzyme-linked immunosorbent assay (ELISA)

ELISAs were conducted to detect IL-6, IL-1ß, IL-8, and TNF-α content in the supernatant. All kits were obtained from R&D Systems (porcine IL-6: #P6000B; porcine CXCL8/IL-8: #P8000, porcine IL-1B/IL-1F2: #PLB00B; porcine TNF-α: # PTA00) and conducted according to the manufacturer’s instructions.

### Statistics

Each experiment was independently repeated at least 3 times. Bar graphs depict mean and standard deviation. Significance was evaluated with Friedman’s ANOVA for three and more groups with post-hoc Wilcoxon’s test. Student’s *t* test was conducted for the comparison of two groups. Statistical analysis has been conducted using Statistica (7.1 StatSoft, Inc.) and Microsoft Excel.

## Results

### Identity of microglia cells: Iba1 staining

**Fig. 1:**
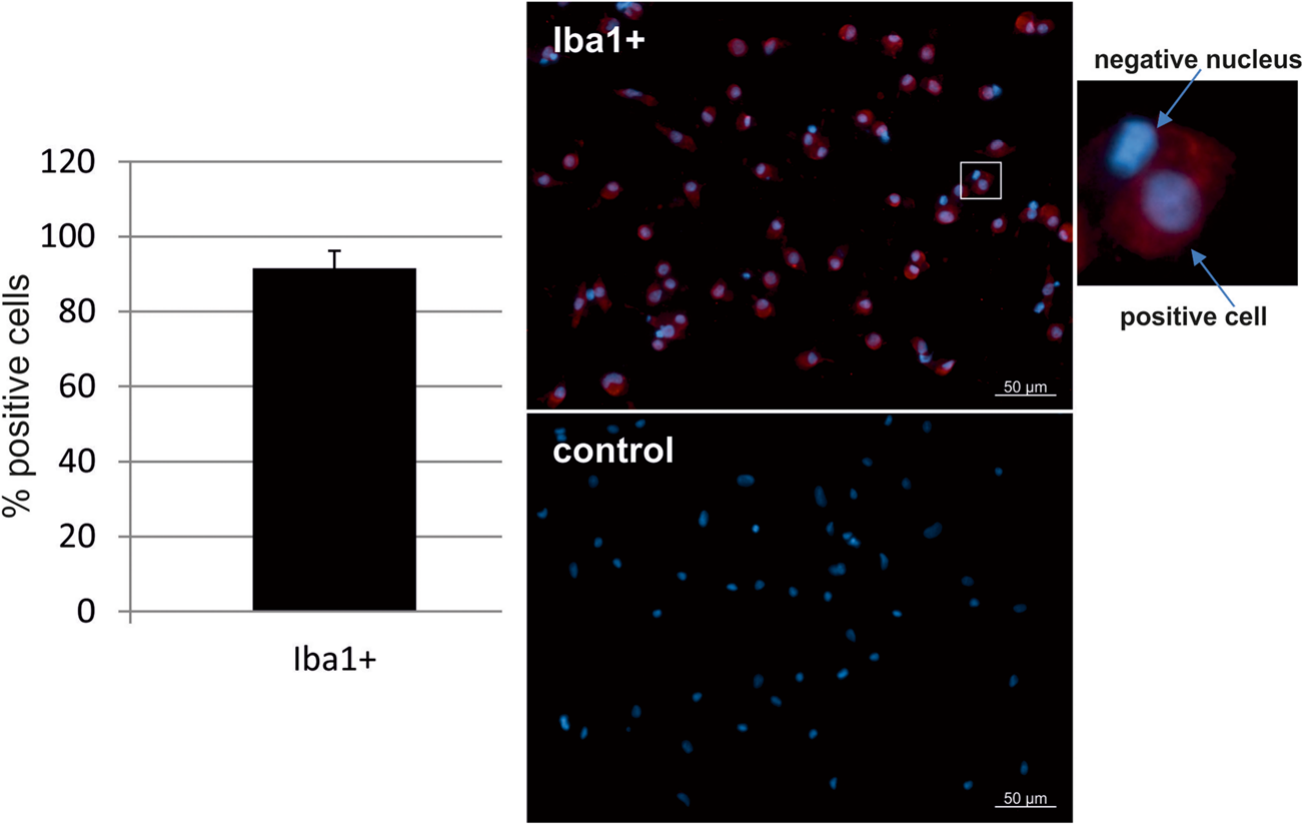
Iba1 staining for the identification of microglia. In immunofluorescence staining, a mean of 91.5% of the cells stained positive for microglia marker Iba1 (*n* = 200 cells, 8 independent experiments). Note the chromatin dense structure of negative nuclei (upper arrow of magnification), indicating that these structures are apoptotic cells, not contaminating cell types

The expression of Iba1 was assessed on microglia seeded on cover slips with a mean of 91.5% positive cells (Fig. 1, 200 cells assessed). Iba1 negative nuclei presented as condensed nuclei with brilliant blue staining indicating cell death [23], rather than contaminating cells.

### Microglia morphology

**Fig. 2:**
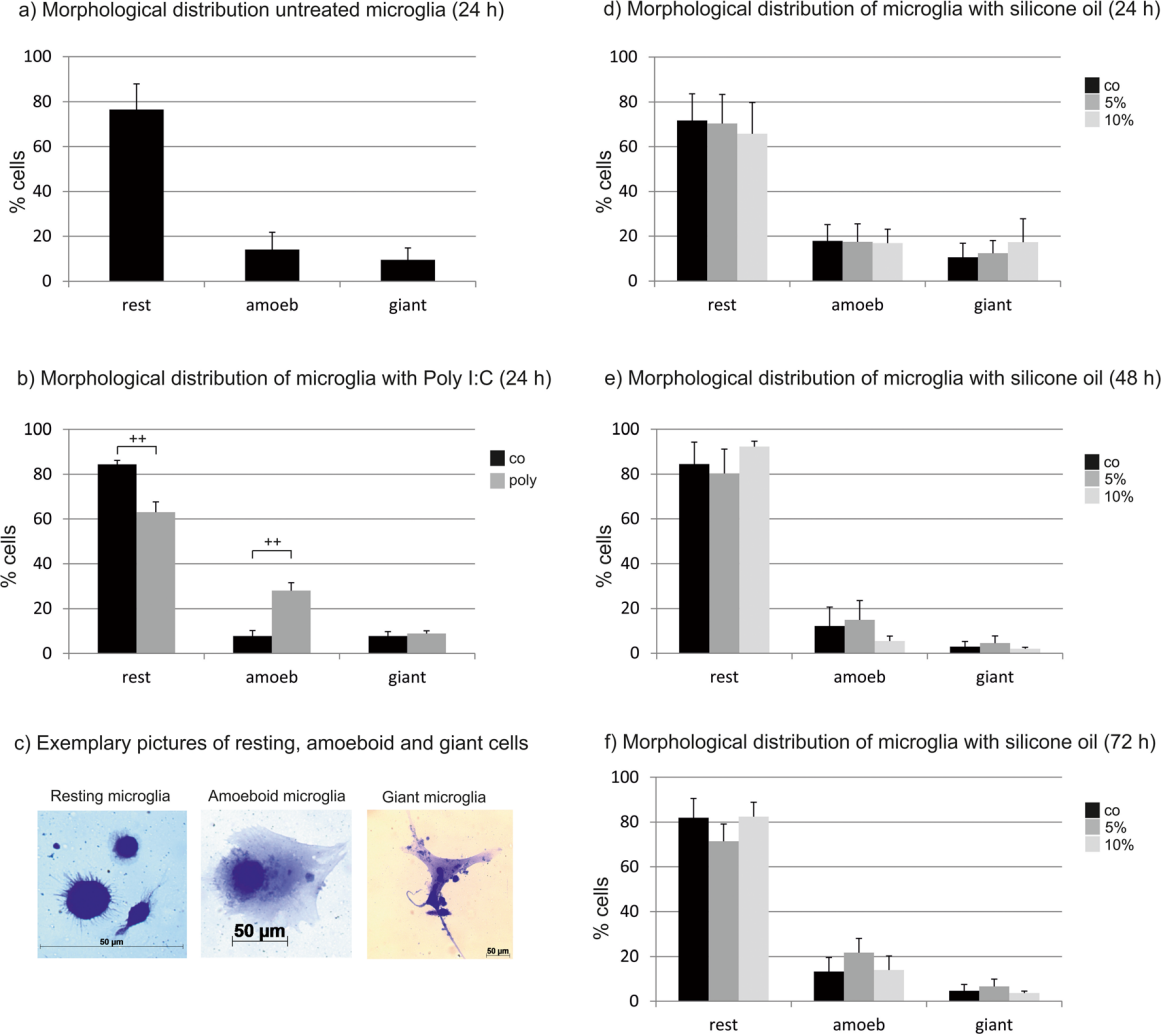
Morphological distribution of microglia cells (resting, amoeboid, giant). **a** Morphological distribution of untreated cells after 24 h of cultivation. **b** Morphological distribution cells treated for 24 h with 100 μg/ml Poly I:C. A significant reduction of resting cells and a significant induction of amoeboid cells can be detected. **c** Resting, amoeboid, and giant cells. **d**–**f** Influence of emulsified silicone oil (5%, 10%) is shown. After 24 h (**d**), 48 h (**e**), and 72 h (**f**), no changes can be observed. Significance was evaluated with Friedman’s ANOVA for a comparison of 3 groups or Student’s *t* test, double positive sign indicates *p* < 0.01. Co, control; rest, resting microglia; amoeb, amoeboid microglia; giant, giant microglia

In untreated control cells after 24 h in culture, 76.52% (± 11.31) of the microglia present as resting, 14.10% (± 7.69) of the microglia present as amoeboid and 9.49% (± 5.30) of the cells present as giant (Fig. 2a, c; 3591 cells assessed). When treated with Poly I:C, the percentage of resting microglia significantly decreased (*p* < 0.01) while the number of amoeboid cells increased (*p* < 0.01) (Fig. 2b; 3087 cells assessed). After treatment with 5% or 10% silicone oil, respectively, no significant changes between the groups (24 h (Fig. 2d; 4980 cells assessed), 48 h (Fig. 2e; 9256 cells assessed) or 72 h (Fig. 2f; 20212 cells assessed)) could be detected in Friedman’s ANOVA (Table 1).

**Table 1:** Morphology of microglia after treatment with 5% or 10% emulsified silicone oil

Time (h)	Morphology	Treatment	Rank	Rank sum	Median	Minimum	Maximum	Friedman
24	Resting	Control	2.25	9	19.53	4.48	24.63	n.s.
		5%	1.75	7	16.21	14.24	23.1	
		10%	2	8	14.42	11.62	27	
	Amoeboid	Control	2.25	9	19.53	4.48	24.63	n.s.
		5%	1.75	7	16.21	14.24	23.71	
		10%	2	8	14.42	11.62	27	
	Giant	Control	2	8	8.3	3.05	20.31	n.s.
		5%	2	8	7.88	5.75	28.48	
		10%	2	8	14.555	6.32	33.96	
48	Resting	Control	1.666667	5	83.25	67.97	94.63	n.s.
		5%	1.333333	4	84.77	65.41	92.79	
		10%	3	9	91.5	89.9	95.67	
	Amoeboid	Control	2.666667	8	9.1	4.29	27.12	n.s.
		5%	2.333333	7	12.5	3.9	25.71	
		10%	1	3	5.59	2.89	8.21	
	Giant	Control	2	6	1.32	0.87	6.63	n.s.
		5%	2.666667	8	3.3	1.84	10.78	
		10%	1.333333	4	1.89	1.44	2.96	
72	Resting	Control	2.2	11	75.8	70.8	94.118	n.s.
		5%	1.4	7	73.68	58.34	82.62	
		10%	2.4	12	86.075	73.32	90.408	
	Amoeboid	Control	1.6	8	17.59	5.11	21.91	n.s.
		5%	2.6	13	20.89	14.06	29.74	
		10%	1.8	9	9.968	6.38	23.89	
	Giant	Control	1.833333	11	4.8325	6.049	0.47	n.s.
		5%	2.666667	16	6.651167	6.805	2.33	
		10%	1.5	9	3.6626	3.8678	2.235	

### Toxicity of silicone oil on microglia cells

**Fig. 3:**
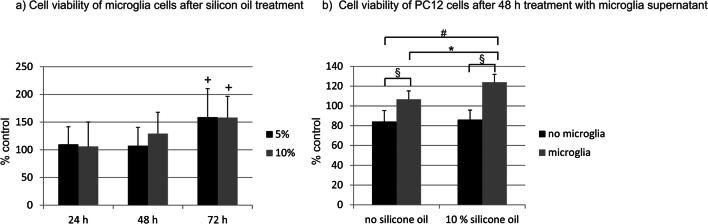
Cell viability testing. **a** Toxicity of emulsified silicone oil on microglia cells was tested after 24, 48, and 72 h for 5% and 10% silicone oil with MTT assay. No toxicity could be detected. Untreated control at the respective time point was set as 100% and is not depicted in the graph. Of note, after 72 h, a significant induction of the signal could be found for 5% (*p* < 0.05) and 10% (*p* < 0.01) compared with untreated control. **b** Cell viability of silicone oil–treated microglia on PC12 cells was tested with WST assay. Microglia were treated for 72 h with emulsified silicone oil, the supernatant was collected, and PC12 cells were incubated with the microglia supernatant for 48 h. In addition, PC12 cells were either treated with microglia medium without silicone oil treatment or directly with emulsified silicone oil. Untreated cells (no microglia, no silicone oil) served as controls. No decline in cell viability could be detected. However, a significant elevation of the PC12 signal could be found when cells were treated with supernatant of microglia exposed to silicone oil, indicating increased metabolic activity. Significance was evaluated with Friedman’s ANOVA and Wilcoxon’s test. **a** Positive sign indicates *p* < 0.05 against untreated control. **b** The section sign indicates *p* < 0.05 comparing cells with and without microglia supernatant treatment. Asterisk indicates *p* < 0.05 comparing cells treated with microglia supernatant with or without silicone oil, and number sign indicates *p* < 0.05 comparing untreated cells with cells treated with supernatant of microglia treated with silicone oil

Toxicity of emulsified silicone oil in microglia cells was tested with an MTT viability assay. No decrease in viability could be found for 5% or 10% silicone oil after 24, 48, or 72 h (Fig. 3a). Of note, a significant increase of MTT signal as detected by Friedman’s ANOVA and post-hoc Wilcoxon’s test after 72 h (*p* < 0.05), suggesting an activation of the cells (Table 2). We additionally tested 1% emulsified silicone oil for 24 h, which also did not display any toxicity (data not shown).

**Table 2:** Viability of microglia after treatment with 5% or 10% emulsified silicone oil

Time (h)	Treatment	Rank	Rank sum	Median	Minimum	Maximum	Friedman	Wilcoxon
24	Control	1.8	9	100	100	100	n.s.	
	5%	2.4	12	126.9	71.4	148.2		
	10%	1.8	9	117.5	49.9	171.6		
48	Control	1.14286	12	100	100	100	n.s.	
	5%	1.714286	12	102.38	51.28	163.13		
	10%	2.571429	18	123.2	73.08	181.8		
72	Control	1.142857	8	100	100	100	*p* < 0.05	
	5%	2.428571	17	125.64	106.25	240.74		*p* < 0.05
	10%	2.428571	17	160	54.93	223.88		*p* < 0.05

### Toxicity of silicone oil–activated microglia cells on neuronal cell line PC12

We tested the toxicity of silicone oil–treated microglia (10% emulsified silicone oil, 72 h) on a differentiated neuronal cell line, PC12 — untreated PC12 cells which served as controls, PC12 cells treated with the supernatant of untreated microglia, and PC12 cells treated with 10% silicone oil. We found no toxicity for either treatment. Of note, treatment with microglia medium, irrespective of the presence of silicone oil, induced a significant increase of the MTS signal (Fig. 3b) in Friedman’s ANOVA and subsequent Wilcoxon’s test, indicating an activating, but not toxic, effect on neuronal cells (Table 3).

**Table 3:** Viability of PC12 cells after 72-h treatment with microglia medium treated with 10% emulsified silicone oil for 48 h

Treatment	Rank	Rank sum	Median	Mini	Maxi	Friedman	Wilcoxon		
							vs (2)	vs. (3)	vs. (4)
Micro/no silicon (1)	2.8	14	109.69	92.64	118.06	*p* < 0.01	*p* < 0.05	*p* < 0.05	n.s.
Micro/10% silicon (2)	4	20	123.07	114.04	138.46			*p* < 0.05	*p* < 0.05
No micro/no silicon (3)	1.3	6.5	86.29	66.55	100				n.s.
No micro/10% silicon (4)	1.9	9.5	84.945	70.57	103.67				

*Mini*, minimum; *Maxi*, maximum; *micro*, microglia

### Uptake of silicone oil into microglia

**Fig. 4:**
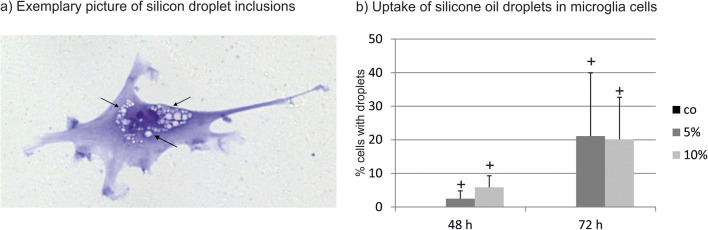
Uptake of silicone oil droplets into microglia. Exemplary picture (**a**) shows clearly visible silicone oil droplets in microglia cells (arrows). Silicone oil droplets were taken up in microglia cells in a time- and concentration-dependent manner (**b**). Untreated microglia that were not treated with silicone oil served as control and displayed few intracellular inclusions according to our definition (72 h, 0.0825%; 48 h, 0.269%). Significance was evaluated with Friedman’s ANOVA and Wilcoxon’s test. Positive sign indicates *p* < 0.05 compared with untreated control

Intracellular silicone oil droplets were clearly detectable in light microscopy (Fig. 4a). We found time-dependent uptake of silicone oil droplets in microglial cells. A total of 12,562 cells were assessed (control 48 h, 2600 cells; 5% silicone oil 48 h, 1387 cells; 10% silicone oil 48 h, 1237 cells; control 72 h, 3310 cells; 5% silicone oil 72 h, 2242 cells; 10% silicone oil 72 h, 1786 cells). Untreated controls displayed few inclusions which fitted our definition of intracellular inclusions (72 h, 0.0825%; 48 h, 0.269%). Friedman’s ANOVA showed a significant difference between the tested groups (48 h, *p* < 0.01; 72 h, *p* < 0.05). In subsequent Wilcoxon’s *t* test, a slight but significant uptake was found after 48 h for 5% and 10%, a stronger uptake was found after 72 h for 5% and 10% (all *p* < 0.05; Table 4) (Fig. 4b).

**Table 4:** Silicone oil droplets in microglia after treatment with 5% or 10% emulsified silicone oil

Time (h)	Treatment	Rank	Rank sum	Median	Minimum	Maximum	Friedman	Wilcoxon
48	Control	1.166667	7	0	0	3.23	*p* < 0.01	
	5%	2	12	4	0	15		*p* < 0.05
	10%	2.833333	17	11	0	34		*p* < 0.05
72	Control	1	6	0	0	0.33	*p* < 0.05	
	5%	2.5	15	15.48	1.84	45.63		*p* < 0.05
	10%	2.5	15	19.71	5.22	44.99		*p* < 0.05

### Cytokine release of microglia after treatment with silicone oil

In order to investigate a possible pro-inflammatory activation of microglia, we investigated the secretion of IL-6, IL-1ß, and TNF-α in ELISA after treatment with 10% silicone oil. Poly I:C was used as a positive control.

**Fig. 5:**
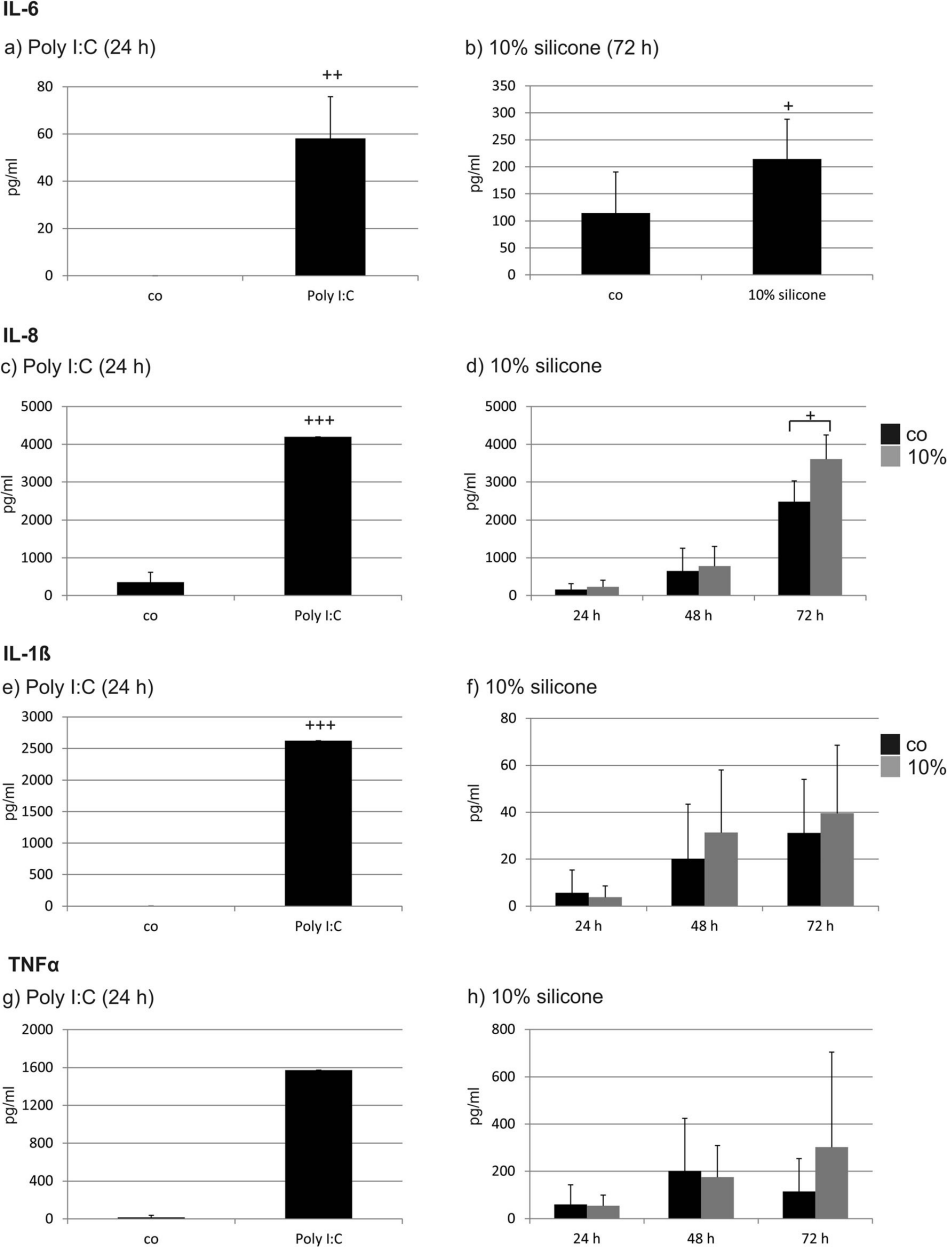
Cytokine excretion by microglia incubated with 100 μg/ml Poly I:C for 24 h (**a**, **c**, **e**, **g**) or 10% silicone for 24 h (**b**, **d**, **f**, **h**) and for 48 and 72 h (**d**, **f**, **h**). Poly I:C induced IL-6 (**a**), IL-8 (**c**), IL-1ß (**e**), and TNF-α (**g**) after 24 h. 10% silicone oil significantly induced IL-6 (b) and IL-8 (d) after 72 h, but not IL-1ß or TNF-α. Significance was evaluated with Student’s *t* test. Positive sign indicates *p* < 0.05; Triple positive sign indicates *p* < 0.001

No secretion of IL-6 could be seen after 24 or 48 h of treatment with silicone oil (data not shown), while Poly I:C induced significant release of IL-6 after 24 h (Fig. 5a). After 72 h, however, both control and silicone oil–treated cells showed secretion of IL-6, displaying a significant increase of IL-6 secretion between 72-h treatment and control (*p* < 0.05).

In contrast, a constitutive secretion of IL-8 could be detected that increased with time. Incubation with Poly I:C for 24 h induced a strong increase of IL-8 secretion (*p* < 0.01). No difference between control and 10% silicone oil was found after 24 or 48 h. However, after 72 h, silicone oil–treated cells showed a significant increase in IL-8 secretion compared with untreated control (*p* < 0.05).

Only very little constitutive IL-1ß could be found after 24 h in microglia supernatant, but it was strongly induced by Poly I:C (*p* < 0.001). Constitutive secretion increased over time but did not change under treatment with 10% silicone after 24, 48, or 72 h. A similar picture could be found for TNF-α, with small amounts being constitutively found after 24 h, a strong induction under Poly I:C, and some increase found over time with no significant changes under treatment with 10% silicone after 24, 48, or 72 h.

## Discussion

In this study, we investigated the effect of emulsified silicone oil on primary retinal microglia. Emulsification is one problem in the use of silicone oil tamponade. In long-term use, silicone oil droplets appear, most likely due to shear stress caused by eye movements. They can usually be found in the angle of the anterior chamber or even in the retina. In selected cases, usually with a history of several surgeries, they can be found in the optic nerve and even the brain [2, 3]. We hypothesized for this study that silicone oil droplets interact with microglia which in turn may lead to microglia-mediated neurotoxicity [24], which might explain the phenomena of the unexplained loss in visual acuity under silicone oil. Emulsification of silicone oil was achieved by sonication in supplemented medium, similar to previously described methods [25].

Inactivated microglia have a typical morphology with a small cell body and numerous extensions, while activated microglia change to an amoeboid state or may even turn into giant cells. Activated microglia may be phagocytic active and secrete cytokines. However, microglia can be activated to different degrees, depending on the stimulus and time scale of activation [19]. Our data clearly shows that silicone oil droplets exert an effect on microglia and that this effect is exercised over 72 h. Due to the culturing condition, we did not exceed 72 h incubation time. We found that microglia took up silicone oil droplets which changed the metabolic status of the cells and induced secretion of IL-6 and IL-8. However, we found no significant changes in cell shape. As a comparison, we used stimulation with Poly I:C, a known activator of the Toll-like receptor (TLR)-3 and inductor of pro-inflammatory responses [15]. The response of our microglia in contact with silicone oil differed from pro-inflammatory-activated microglia treated with Poly I:C. While Poly I:C induced a general mobilization of cytokines, with an induction of IL-6, IL-8, IL-1ß, and TNF-α, the induction by silicone oil could only be detected for IL-6 and IL-8. It is of interest that the induction of cytokines is not accompanied by a change in microglial morphology. This could be an indication that the silicon droplet uptake is not inducing a full-scale activation of the microglia but induce only a partial response. This would be in accordance with differentiated cytokine release we can see, with IL-1ß and TNFα not being induced by silicon droplets. Our data suggests that silicone oil induces a differentiated effect. IL-1ß and TNF-α have been described to be neurotoxic [26], and their release by activated microglia can exert a direct toxic effect on neurons [27]. IL-6, on the contrary, can be neuroprotective, depending on receptor and signaling mechanisms [28, 29]. IL-8 primarily induces chemotaxis [30], but neurotoxic effects have been described for IL-8 as well [31]. Recently, elevated IL-8 levels have been correlated with acute vision loss and persistent dysfunction after acute optic neuritis [32]. Whether this mechanism is also relevant in the cases with unexplained loss in visual acuity after the use of silicone oil tamponade is speculative. A further evaluation of the levels of IL-8, e.g., in aqueous humor of patients with silicone oil tamponade in correlation with vision loss, would therefore be of high interest.

We further evaluated whether silicone oil–treated microglia exerted toxic effects on neuronal cells. Supernatants of silicone oil–treated microglia clearly did not induce cell death in differentiated PC12 cells, elevating the signal in the WST assay. As WST measures metabolic activity, our data clearly indicate that microglia exert an effect on neuronal cells, which, however, does not result in neuronal cell death, at least not in the measured time frame. The increase in the viability signal found both for microglia and PC12 cells could also indicate an activation of the cells in order to enable them to be more stress resistant, therefore, protective effects of the microglia on the neuronal cells could also be possible. Further research is needed to address this aspect.

Patients are treated with silicone oil for several months. We could show in our study that microglia react to silicone oil when it is in an emulsified state given an appropriate exposition time. Our data did not show any toxicity of microglia on neuronal cells after a 3-day exposure. Translated to the in vivo situation, this could imply that no acute toxicity is mediated by microglia in silicone oil–treated patients in the early period. This corresponds with the clinical data that usually do not see acute toxicity. It is difficult to transfer the time frame of cell cultures to the clinical time frame. Visual acuity loss usually occurs in the intermediate phase (6 weeks to 6 months) of the silicone oil tamponade. Our data would suggest that a long-term interaction of microglia with silicone oil might induce long-term pro-inflammatory changes and a long-term secretion of pro-inflammatory cytokines. As described in the literature, neurotoxicity of microglia usually develops when a pro-inflammatory stimulus cannot be eliminated [33]. An additional aspect that has to be considered is a priming of microglia. When primed, microglia will react much faster and more intense to an additional stimulus [19]. Translated to the in vivo situation, this could imply that patients treated with silicone oil for an extended time period may experience an otherwise milder noxious stimulus in a more neurotoxic manner.

Our data shows that retinal microglia react to emulsified silicone oil and our study clearly warrants further research on the long-term effect of silicone oil tamponades on retinal microglia and its consecutive effect on retinal neurons.
